# We need to talk about social class: Why theories of social class matter for understanding inequities in palliative and end-of-life care

**DOI:** 10.1177/02692163241296478

**Published:** 2024-10-30

**Authors:** Andy Bradshaw, Naomi Richards, Jamilla A. Hussain, Joanna M. Davies

**Affiliations:** 1 King’s College London, Cicely Saunders Institute of Palliative Care, Policy and Rehabilitation, London, UK; 2 University of Glasgow, End of Life Studies Group, School of Interdisciplinary Studies Rutherford/McCowan Crichton Campus, Dumfries, UK; 3 Bradford Teaching Hospitals NHS Foundation Trust Bradford, Bradford, UK; 4 Bradford Institute for Health Research Bradford, Bradford, UK

**Keywords:** Social class, Bourdieu, end-of-life inequities, inequalities, intersectionality, classism

## The ‘equity turn’ in palliative and end-of-life care

Palliative and end-of-life care is undergoing an ‘equity turn’ in which funders, researchers and health services are increasingly recognising how access to and outcomes of care are experienced inequitably.^1^ Inequities represent ‘differences in health that are not only unnecessary and avoidable’ but ‘unfair and unjust’.^2^ A crucially important social determinant of health (and of dying well) that has largely been left out of these conversations is social class. The purpose of this editorial is to contribute to the equity turn by making the case for how social class theory should inform research on equity in palliative and end-of-life care.

## Social class at the end of life

### Understanding social class

At its core, social class is hierarchical and relational. Whilst class has most commonly been described and applied in the context of British society, it has relevance to all societies that are socially stratified and hierarchical. The caste system in India is another well-known example of a social stratification system. Class also intersects with other characteristics to produce multiple advantages or disadvantages, and in some contexts class status has been tied to race and ethnicity, for example in the colonial Casta system in Latin America and Apartheid in South Africa.

Internationally, social class provides a lens through which to understand how social groups relate to and interact with one another, how discrimination and prejudice occur as a result of hierarchical class relations, and ultimately how wealth and power is distributed in a society. To properly understand contemporary class dynamics and prejudices, Bourdieu’s influential cultural class analysis^3^ encourages us to avoid treating class as synonymous with socioeconomic position or reducing it to single dimensions of occupation, education, income, and wealth. Instead, to comprehensively understand class requires awareness of its symbolic, cultural, and social features. Bourdieu’s theory provides opportunity to do this through his concepts of *fields* (institutional contexts, socio-cultural codes and etiquette), *habitus* (social biography and learned dispositions including how people speak, feel and behave) and *capital* (economic, social and cultural).

### Acknowledging class inequities and discrimination exist at the end of life

A crucial step in understanding and addressing inequities at the end of life is to acknowledge that class discrimination and prejudice exist, and that unequal distributions of wealth and power impact end-of-life choices, experiences and outcomes. Bourdieu’s concepts of field, habitus and capital provide tools that can help to deconstruct the ways class structures work to reinforce class discrimination within palliative and end-of-life care specifically. Evidence shows that those with lower socioeconomic position (thus reduced economic capital) are less likely to access specialist palliative care and more likely to use hospital-based care in the last months of life.^4^ But the ways in which class structures function at the end of life exceed economic issues; prejudice, discrimination and marginalisation related to social class occur more subtly through symbolic, social and cultural mechanisms.

From a Bourdiesian perspective, the ‘field’ of palliative and end-of-life care practice and research privileges those who possess dominant forms of habitus and social and cultural capital. For example, traditionally, services have tended to be designed for ‘typical’ patients who possess multiple privileges. These include living in secure, warm housing, being able to pay for out-of-pocket healthcare and additional illness-related costs, and having more confidence in navigating complicated health and social care systems.^1^ The class composition of academics, doctors and senior nurses is also skewed towards the dominant classes.^5,6^ Consequently, middle-class aesthetics, worldviews and dispositions are the dominant framework through which end-of-life issues are conceptualised, investigated, represented and challenged.^5^ This is problematic because it risks devaluing and othering the experiences, preferences and aesthetic values of social groups which do not align with the socio-cultural norms of the dominant classes. It also means more subtle class-based discrimination – what Bourdieu calls ‘symbolic violence’ – goes unchecked.

Examples of classism in palliative and end-of-life care are present in research and policy whereby narratives of individual choice and personal responsibility are prioritised above structural explanations for understanding and addressing class inequalities.^7^ Symbolic violence is also present in the classist language, stereotypes and stigmatisations used by health professionals and academics, some of which have been documented in qualitative research. Examples include homeless people seeking pain relief being automatically labelled by healthcare professionals as ‘drug seeking’,^8^ structurally vulnerable groups having care discontinued due to their living situation being deemed too ‘risky’,^9^ and people living in poverty being referred to as ‘unkempt’, living in ‘dirty’ housing, and needing education on better ‘managing their money’.^10^ Whilst these examples may be viewed as ‘objective’ (or even sympathetic) observations about the cleanliness of a person or their living conditions, this language is attached to broader and more subtle forms of class demonisation and classist tropes that label the working-classes, the places they live, and culture as problematic, stupid, lacking, vulgar and repulsive.^11^

## What does this mean for future research, researchers, and health professionals?

Only by recognising the ways that class structures operate can researchers and health professionals begin to understand and address the class-based discrimination, prejudice and disadvantage experienced by patients and carers towards the end of life. Whilst there is a great need for more research in this area, *who* conducts research and *how* it is conducted is equally as important as *what* we research. The following are suggestions for how we can start to incorporate a critical awareness of social class into palliative and end-of-life care research, policy, practice and funding:

• ***Centring at the margins.*** It is essential that future work seeks to redress historical imbalances by ‘centring at the margins’ and adopting the principle of proportionate universalism.^12^ Committing to proportionate universalism means recognising that, whilst access to and quality of palliative and end-of-life care should be improved for all social classes, to achieve this equitably and fairly, the scale and intensity of research, practice and funding needs to be shifted proportionally based on degree of disadvantage. This approach means we can continue to raise standards for all whilst simultaneously closing class gaps. In practice, this means finding meaningful ways to work in partnership with underserved groups to better understand how to design and deliver services and care in appropriate and culturally-responsive ways. This includes working with the traditional working classes and the ‘precariat’; those with the least economic, social and cultural capital, often living and working precariously without stable occupations or sufficient social protection. It also means acknowledging class as a critical aspect of intersectionality. Doing this allows us to explore how social class intersects with other systems of power and oppression such as racism, sexism, ageism and ableism, leading to inequities in end-of-life experiences and outcomes.
• ***A politically-informed, equity-driven agenda.*** Commitment to equity-informed research requires researchers to critically analyse how the inequitable distribution of power and wealth perpetuates inequities towards the end of life, just as it does across the life course. This cannot be done apolitically. Instead, it is essential that researchers become politicised in deconstructing the root political, economic, institutional and socio-cultural foundations that lead to structural class (and other intersectional) inequities towards the end of life.^1^ Researchers and health professionals working in palliative and end-of-life care cannot be expected to remove social class structures and hierarchies as individuals. However, through long-term collective action (between researchers, research teams, governing bodies, representative groups, funding bodies and organisations), we can and should use our positions and spheres of influence to engage with social class theory in our research, practice and personal politics. In doing so, we can work together to highlight the implications of, and seek to challenge and mitigate, class inequities within our institutions, organisations, services, departments and personal lives.
• **Using social class theory to focus on structural causes and solutions.** Currently, research exploring inequities at the end of life is dominated by pragmatic measurement-based approaches that often lack underpinning theory.^1^ Whilst useful in quantifying economic class inequities, these approaches often fail to capture the fundamental structural, cultural and relational mechanisms through which social class operates towards the end of life. As such, they risk misattributing causes and misdirecting interventions which have the potential to widen class inequities by focusing on personal responsibility and individual choice without recognising the structural constraints that people operate within or that choices are influenced by class issues. Social class theory can help to refocus attention on the underlying structural causes of inequities within palliative and end-of-life care and support researchers to carefully consider which measures they select and on what theoretical basis. Crucially, we must also move beyond only using measurement-based approaches to provide a ‘three-dimensional understanding’ of class at the end of life and test innovative equity-informed interventions.^5^ In-depth qualitative approaches provide opportunity to do this through focusing on the social, cultural, political, relational and economic factors that contribute to classism at the end of life.
• **Critical Participatory Action Research approaches.** In working towards a more political, theoretical and equity-informed approach to scholarship, researchers can draw upon critical participatory action research.^13^ These research designs are: (i) ***critical***, by using critical class theories to ask questions about how end-of-life experiences are impacted by classist structures and inequitable distributions of power and wealth; (ii) ***participatory***, by engaging in democratic knowledge production that works *with* working class people, as opposed to research done on and to ‘others’. In using participatory approaches, research needs to move away from deficit models of thinking which frame the working classes as in need and helpless. Rather, they should be grounded in strength-based approaches which acknowledge and celebrate the capacities and contributions that working class forms of capital bring to palliative and end-of-life care research and practice; and (iii) ***action-based***, using rigorous research to invoke and advocate for direct social change and action at individual, team, service, organisational and policy levels.
